# Supplementary material for: From sequence to enzyme mechanism using multi-label machine learning
Source: BMC Bioinformatics. 2014 May 19;15:150. doi: 10.1186/1471-2105-15-150 (PMC4229970; doi:10.1186/1471-2105-15-150)
Supplement: Additional file 2 — Java code of ml2db. Additional file ml2db_code.tar.gz contains the Java source code to run the multi-label machine learning experiments and save the results to database. The code’s Javadoc is included. [file 1471-2105-15-150-S2.zip › additional file 2/ml2db/ml2db/doc/main/ml2db/al/utility/pickers/byfrequency/NearestNeighboursByFreqUtility.html]

NearestNeighboursByFreqUtility


JavaScript is disabled on your browser.


- Overview
- Package
- Class
- Use
- Tree
- Deprecated
- Index
- Help

- Prev Class
- Next Class

- Frames
- No Frames

- All Classes

- Summary:
- Nested |
- Field |
- Constr |
- Method

- Detail:
- Field |
- Constr |
- Method


main.ml2db.al.utility.pickers.byfrequency

## Class NearestNeighboursByFreqUtility

- java.lang.Object
- - main.ml2db.al.utility.UtilityMetric
  - - main.ml2db.al.utility.pickers.RandomPicker
    - - main.ml2db.al.utility.pickers.byfrequency.ByFrequencyPicker
      - - main.ml2db.al.utility.pickers.byfrequency.NearestNeighboursByFreqUtility

- All Implemented Interfaces:
  :   Picker

  ---

    

  ```
  public class NearestNeighboursByFreqUtility
  extends ByFrequencyPicker
  ```

  Class

  Version:
  :   13 May 2012

  Author:
  :   Luna De Ferrari ldf(at)st-andrews.ac.uk or
      luna.deferrari@st-andrews.ac.uk

- - ### Field Summary

    Fields

    | Modifier and Type | Field and Description |
    | --- | --- |
    | `static java.lang.String` | `NEIGHBOURHOOD_BASE_NAME` |

    - ### Fields inherited from class main.ml2db.al.utility.pickers.RandomPicker

      `m_debug`
  - ### Constructor Summary

    Constructors

    Constructor and Description

    `NearestNeighboursByFreqUtility()`
  - ### Method Summary

    Methods

    | Modifier and Type | Method and Description |
    | --- | --- |
    | `uk.ac.standrews.utils.main.maputils.OneToManyMap<java.lang.String,java.lang.String>` | `generateInstanceToFeaturesMap()` |
    | `java.lang.String` | `getCurrentNeighbourhoodName()` |
    | `LinearNNSearchSkipInstanceItself` | `getLinearNNSearch()` |
    | `java.lang.String` | `getMetricName()` |
    | `static java.lang.String` | `getSerialisationFilePath()` |
    | `void` | `initialise(IdMultiLabelInstances unlabelled)` |
    | `void` | `refresh(IdMultiLabelInstances labelled, IdMultiLabelInstances unlabelled, IdInstance queryInstance)` Refresh the set of labelled and unlabelled instances |
    | `void` | `setCurrentNeighbourhoodName()` |
    | `void` | `setTest(boolean test)` |

    - ### Methods inherited from class main.ml2db.al.utility.pickers.byfrequency.ByFrequencyPicker

      `calculateFrequency, getFeaturesByDescendentFrequency, getFrequencyToFeatureMap, initialise, orderAttsetByDescFreq, setFeaturesByDescendentFrequency, setFrequencyToFeatureMap`
    - ### Methods inherited from class main.ml2db.al.utility.pickers.RandomPicker

      `getInstanceToFeaturesMap, getQueries, getQuery, getStep, getUtility, isDebug, isTest, setDebug, setFeatureToInstanceMap, setStep`
    - ### Methods inherited from class main.ml2db.al.utility.UtilityMetric

      `getUnlabelled, setUnlabelled`
    - ### Methods inherited from class java.lang.Object

      `equals, getClass, hashCode, notify, notifyAll, toString, wait, wait, wait`

- - ### Field Detail


    - #### NEIGHBOURHOOD\_BASE\_NAME

      ```
      public static final java.lang.String NEIGHBOURHOOD_BASE_NAME
      ```

      See Also:
      :   Constant Field Values
  - ### Constructor Detail


    - #### NearestNeighboursByFreqUtility

      ```
      public NearestNeighboursByFreqUtility()
      ```
  - ### Method Detail


    - #### generateInstanceToFeaturesMap

      ```
      public uk.ac.standrews.utils.main.maputils.OneToManyMap<java.lang.String,java.lang.String> generateInstanceToFeaturesMap()
      ```


    - #### getCurrentNeighbourhoodName

      ```
      public java.lang.String getCurrentNeighbourhoodName()
      ```

      Returns:
      :   m\_currentNeighbourhoodName


    - #### getLinearNNSearch

      ```
      public LinearNNSearchSkipInstanceItself getLinearNNSearch()
      ```

      Returns:
      :   m\_linearNNSearch


    - #### getMetricName

      ```
      public java.lang.String getMetricName()
      ```

      **Specified by:**
      :   `getMetricName` in class `ByFrequencyPicker`

      Parameters:
      :   `freqToInstancesMap` - m\_freqToInstancesMap to set


    - #### initialise

      ```
      public void initialise(IdMultiLabelInstances unlabelled)
      ```


    - #### refresh

      ```
      public void refresh(IdMultiLabelInstances labelled,
                 IdMultiLabelInstances unlabelled,
                 IdInstance queryInstance)
      ```

      **Description copied from class: `UtilityMetric`**

      Refresh the set of labelled and unlabelled instances

      **Overrides:**
      :   `refresh` in class `RandomPicker`
      :   `unlabelled` - protected abstract void prepareQueryInstancesList(
          IdMultiLabelInstances unlabelled);


    - #### setCurrentNeighbourhoodName

      ```
      public void setCurrentNeighbourhoodName()
      ```

      Parameters:
      :   `currentNeighbourhoodName` - m\_currentNeighbourhoodName to set


    - #### setTest

      ```
      public void setTest(boolean test)
      ```

      **Overrides:**
      :   `setTest` in class `RandomPicker`

      Parameters:
      :   `test` - m\_test to set


    - #### getSerialisationFilePath

      ```
      public static java.lang.String getSerialisationFilePath()
      ```

      Returns:


- Overview
- Package
- Class
- Use
- Tree
- Deprecated
- Index
- Help

- Prev Class
- Next Class

- Frames
- No Frames

- All Classes

- Summary:
- Nested |
- Field |
- Constr |
- Method

- Detail:
- Field |
- Constr |
- Method
